# Need for evidence synthesis for quality control of healthcare decision-making

**DOI:** 10.3126/nje.v13i3.61004

**Published:** 2023-12-23

**Authors:** Brijesh Sathian, Edwin van Teijlingen, Israel Junior Borges do Nascimento, Mahalaqua Nazli Khatib, Indrajit Banerjee, Padam Simkhada, Russell Kabir, Hanadi Al Hamad

**Affiliations:** 1 Geriatrics and long term care department, Rumailah Hospital, Hamad Medical Corporation, Doha, Qatar; 2 Centre for Midwifery& Women’s Health, Bournemouth University, Bournemouth, UK; 3 Division of Country Health Policies and Systems, World Health Organization Regional Office for Europe, Copenhagen, 2100, Denmark; 4 Division of Evidence Synthesis, DMIHER, Wardha, India; 5 Sir Seewoosagur Ramgoolam Medical College, Belle Rive, Mauritius; 6 School of Human and Health Sciences, University of Huddersfield, Huddersfield, UK; 7 School of Allied Health, Anglia Ruskin University, Essex, UK

**Keywords:** Evidence synthesis, systematic reviews, scientific research, decision-makers

## Abstract

Systematic reviews that are out-of-date delay policymaking, create controversy, and can erode trust in research. To avoid this issue, it is preferable to keep summaries of the study evidence. Living evidence is a synthesis approach that provides up-to-date rigorous research evidence summaries to decision-makers. This strategy is particularly useful in rapidly expanding research domains, uncertain existing evidence, and new research that may impact policy or practice, ensuring that physicians have access to the most recent evidence. Addressing global challenges – ranging from public health crises to climate change or political instability - requires evidence-based judgements. An obsolete, biased, or selective information poses risks of poor decisions and resource misallocation. The relatively nascent practice of living evidence proves invaluable in maintaining continuous interest and team engagement. The concept of living evidence has been particularly relevant during the COVID-19 pandemic due to the rapidly evolving nature of the virus, the urgent need for timely information, and the continuous emergence of new research findings. Although the COVID-19 pandemic accelerated the adoption of evidence systems, researchers and funders of research should rigorously test the living-evidence model across diverse domains to further advance and optimize its methodology.

## Background

Evidence synthesis involves gathering and synthesizing data from relevant studies to address a specific and precise question or set of questions. This ensures that decision-makers understand all the existing evidence, diminishing the risk of making decisions rooted in incomplete or biased information. Evidence synthesis helps build a holistic body of knowledge, which serves as the foundation for decision making in areas such as health, education, and global development. With his 1753 published book on scurvy [1], James Lind, a Scottish physician, was a pioneer in systematic evidence synthesis, a field in which he made a substantial contribution. Lind’s book was reprinted by Cambridge University Press a decade ago [2]. A more recent contribution to evidence synthesis was by another Scottish physician Archibald ‘Archie’ Cochrane who published Effectiveness and efficiency: Random reflections on health services in 1972 [3],

Moreover, a deeper exploration of the methodologies established by university departments and international bodies, such as the Cochrane Collaboration, the Campbell Collaboration, the Grading of Recommendations, Assessment, Development, and Evaluation (GRADE) Working Group, the World Health Organization (WHO), the National Institute for Health and Care Excellence (NICE), the Joanna Briggs Institute (JBI), and the Agency for Healthcare Research and Quality (AHRQ)have produced scientific methodologies for evidence synthesis, which are now used by policy-makers and practitioners alike. Though the standardization and improvement of methodologies for evidence synthesis by these organizations promote the use of high-quality evidence in healthcare decision-making on a global scale, the methodology applicable to major global concerns such as climate change, lifestyle modification, biodiversity loss, healthy ageing, energy transitions, antibiotic resistance, and poverty reduction is frequently under-resourced, disorganized, and outdated. Emphasizing an interdisciplinary approach, these methodologies amalgamate findings from disparate fields to address multifaceted global challenges, which are sometimes referred to as ‘wicked problems’. This adaptability allows evidence synthesis to serve as a linchpin in comprehensively tackling complex issues by integrating insights across diverse domains.

Currently, the rigorous methodology and comprehensive approach of systematic reviews and meta-analyses are heavily used in evidence-based health-care decisions, making them an important tool for appraising and synthesizing research findings and policymaking. Extending these methodologies to address interdisciplinary challenges serves as a testament to their potential in offering comprehensive solutions beyond the confines of healthcare.

Systematic reviews are important for synthesizing existing evidence and evaluating the quality of such evidence from individual studies. It assesses the likelihood of bias, identified difficulties that erode trust in what is known, and provided a glimpse of flaws in design, small sample sizes, lack of registration, and inconsistent reporting. Systematic reviews provide a basis for developing clinical guidelines by employing rigorous methods for literature search, study selection, and data synthesis and offering a more reliable, comprehensive, and unbiased summary of available evidence.

Original research is frequently prioritized over evidence synthesis in evaluation systems, particularly in the clinical domain. However, as proven during the pandemic, evidence synthesis can be useful for policy and decision-making by offering high-quality, scientifically meaningful contributions in rapidly evolving circumstances.

PRISMA is a technique for evaluating systematic reviews and meta-analyses in fruitful research fields [4]. Peer review and high methodological rigor were used to ensure that the review fulfilled appropriate requirements. The authors must use PROSPERO [5], first to check if their review question is not already addressed elsewhere, and secondly, to register their reviews to help others to find it. In addition, they must complete and submit a PRISMA flow diagram [4], which clearly shows the number of records detected, inclusions, and exclusions.

The COVID-19 pandemic underscored the vulnerabilities in the evidence pipeline, leading to the rapid emergence of low-quality guidelines, position statements, and protocols, including the use of remdesivir, an intravenous therapy initially intended for the Ebola virus. The pandemic disrupted the evidence pipeline, causing a shift in the body of evidence that usually excluded current primary studies and was quickly outdated [6].

Systematic reviews that are out-of-date delay policymaking, create controversy, and can erode trust in scientific research. To avoid this issue, it is preferable to keep summaries of the study evidence. Living evidence is a synthesis approach that provides up-to-date rigorous research evidence summaries to decision makers. This strategy is particularly useful in rapidly expanding research domains, uncertain existing evidence, and new research that may impact policy or practice, ensuring that physicians have access to the most recent evidence [7]. To identify new research and stay abreast of the latest developments, researchers have developed mechanisms such as periodic upgrades, database monitoring, use of preprint servers, institutional repositories, subscribing to Table of Contents (TOC) alerts etc. Natural language processing, machine learning, and crowdsourcing technologies have enabled such projects. The experiments were carried out in cycles, following well-established techniques for high-quality synthesis. To ensure that policymakers, practitioners, the general public, and the media, are aware of relevant information, living recommendations are constantly updated with research findings. This method proved critical during the COVID-19 pandemic because it helps to maintain vaccine effectiveness while simultaneously addressing concerns.

The UK NICE has declared that living guidelines are a key component of its five-year improvement agenda [8]. The substantial adoption of living systematic reviews by these influential bodies underscores the credibility and utility of this approach in shaping evidence-based practices and policies. Living evidence is critical for decision making, which necessitates a cultural shift among funders and politicians. Engaging stakeholders and end-users in the conceptualization and execution of living evidence initiatives, including various communities, vocational training programs, and disease treatment standards fosters a participatory approach. Such involvement ensures the relevance and applicability of synthesized evidence in addressing diverse societal needs and challenges.

A systematic review is critical for increasing the availability of living evidence, but it typically involves over 200 person-hours of manual labour. Natural language processing, machine learning, crowdsourcing, FAIR (Findable, Accessible, Interoperable, Reusable) data principles, alongside specialized software solutions such as Covidence^®^ and MAGICapp^®^ can significantly streamlines the workflow efficiency and enhances cost-effectiveness. These tools reduce the time required by half. Enhancing metadata and adhering to FAIR practices for open and machine-readable research data within publications significantly enhances the quality and accessibility of synthesized evidence. Whilst, low-quality reviews, which are widespread in systematic reviews, may result in research wastage. Researchers and journals frequently pursue rapid systematic reviews to increase the number of publications and citations. Prioritizing high-quality living systematic reviews and guidelines, fostering collaborative initiatives to minimize redundancy, and elevating research quality standards are essential steps to steer away from the pursuit of quantity over quality in published reviews.

Esteemed academic publishers like the *Cochrane Database of Systematic Reviews*, Journals like Systematic Reviews, published by BioMed Central, *F1000, BMJ*, and *Annals of Internal Medicine* have shown adaptability by accommodating regular updates to systematic reviews and guidelines. Mostly minor, these updates have been seamlessly integrated ensuring continuous ties across various versions. Incorporating minor revisions within the original publication or as addenda allows for transparent documentation, while substantial modifications warrant the generation of a fresh article version and bibliographic listings.

The research community needs to reconsider the version of the record and improve the incentives for authors and peer reviewers. Evaluating the influence of new dissemination models, determining optimal instances when evidence changes should drive implementation decisions, and ensuring the integration of credible evidence into decision-making processes are crucial considerations. Living evidence can help jump-start implementation operations. The integration of living evidence into implementation strategies fosters a responsive, adaptable, and evidence-driven approach. Aligning outputs, such as healthcare recommendations and climate-change mitigation models, with robust data systems bolsters the support available to physicians, facilitating informed decision-making. Addressing global challenges – ranging from public health crises to climate change or political instability - requires evidence-based judgements. An obsolete, biased, or selective information poses risks of poor decisions and resource misallocation. The relatively nascent practice of living evidence proves invaluable in maintaining continuous interest and team engagement. The concept of living evidence has been particularly relevant during the COVID-19 pandemic due to the rapidly evolving nature of the virus, the urgent need for timely information, and the continuous emergence of new research findings. Although the COVID-19 pandemic accelerated the adoption of evidence systems, researchers and funders of research should rigorously test the living-evidence model across diverse domains to further advance and optimize its methodology.
